# Visual Snow Syndrome: Therapeutic Implications

**DOI:** 10.3390/jcm14176070

**Published:** 2025-08-27

**Authors:** Kenneth J. Ciuffreda, Daniella Rutner

**Affiliations:** 1 Department of Biological and Vision Science, College of Optometry, State University of New York, New York, NY 10036, USA; 2 Department of Clinical Education, Vision Rehabilitation Service, College of Optometry, State University of New York, New York, NY 10036, USA

**Keywords:** visual snow, visual snow syndrome, treatment, neurology, neuro-optometry, clinical medicine

## Abstract

Visual snow and its syndrome represent a relatively new and enigmatic neurological condition affecting the human sensory, motor, and perceptual systems. In this narrative review, first an overview of the condition and its basic characteristics and demographics are presented. Then, the six therapeutic approaches that have been attempted over the past decade are detailed by a simple discussion of the problem with the patient, medications, special chromatic tints, oculomotor training, visual noise adaptation, and environmental changes, which have met with varying degrees of success. Thus far, chromatic tints and oculomotor training appear to be the most successful.

## 1. Introduction

The neurological condition of visual snow (VS), and its more encompassing syndrome (VSS), have been, and remain, an enigma in clinical medicine and beyond [1]. It transcends many specialty disciplines: neurology, neuro-ophthalmology, ophthalmology, physiatry, neuro-psychology, neuro-optometry, and more [1,2]. The primary objective of this focused narrative review is to provide the clinical medicine doctor and others in the clinical field (e.g., neurology and psychology) with a brief, but broad and current, comprehensive overview of current and future possible treatment for those with VS/VSS.

The condition of VS was likely first described in 1944 as a visual side-effect in some patients prescribed digitalis for heart problems [3]. It was “rediscovered” in 1995 as a symptom in many with migraines [4]. However, VS was later found to be distinct from migraines, and thus migraines represent a common (65%), but comorbid, condition in those with VS [5,6]. Its etiology is varied, including concussion, medications, sequelae of brain surgery, and stroke, and is frequently unknown [1,7,8,9].

**Figure 1:**
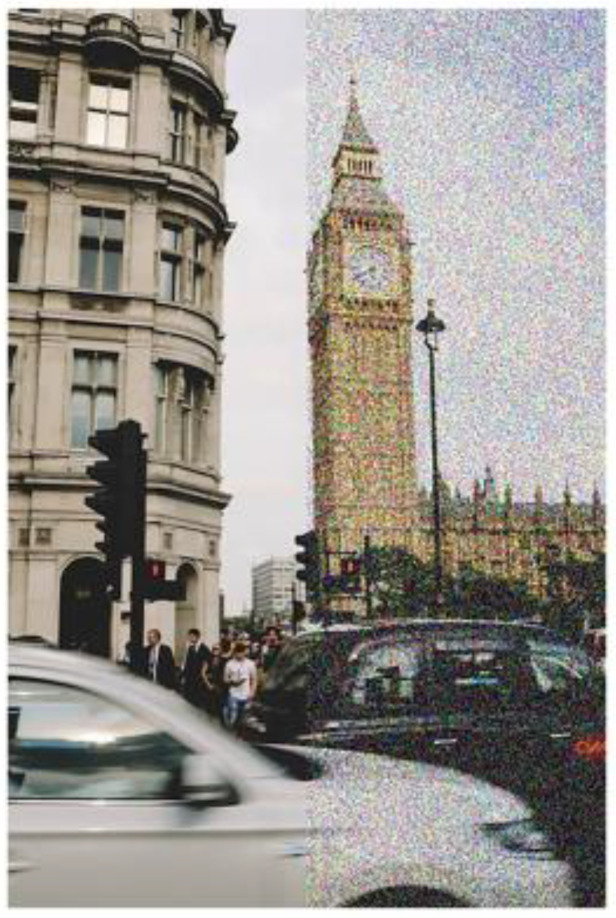
Simulation of normal (**left**) and visual snow (**right**) scene. With permission, from [1]. Original Figure 1.

**Figure 2:**
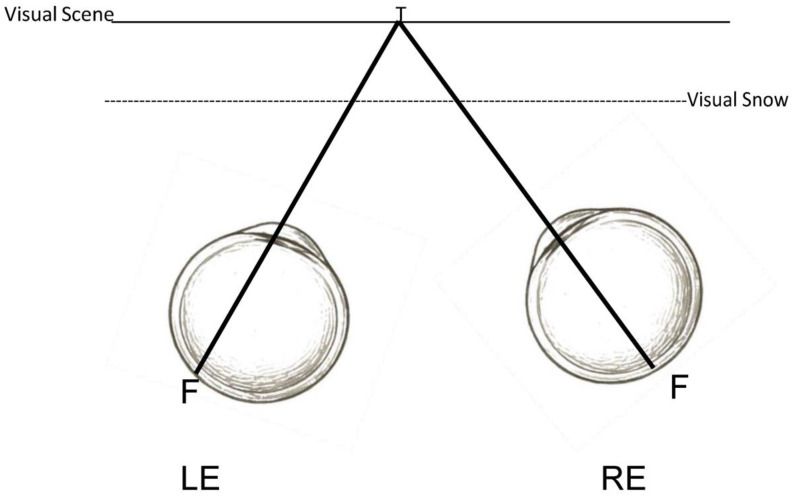
Binocular perception of the visual scene with VS overlay in the foreground (top view. LE = left eye, RE = right eye). Symbols T = target, F = fovea, LE = left eye, RE = right eye.

What is VS? It refers to the visuo-perceptual abnormality of seeing a pixelated overlay of dynamic “visual noise”, or moving dots, over one’s entire visual field [1,2,3,4,5,6] (Figure 1). It is somewhat akin to the image that occurs on a detuned television screen [10]. Thus, there are two depth planes of visual context: the foreground proximal plane of the VS itself, and the background distal plane of the visual scene (Figure 2). VS has an estimated prevalence of 3.7% in the U.K. population [11]. VS is typically constantly present, even with the eyes closed and in the dark, and is monochromatic in nature (80%), but it can also occur transiently and be achromatic in nature (20%) [1]. If constantly present, then it can become a long-term problem of 7 years or more [12].

In contrast, there is the broader visual snow syndrome (VSS) with its constellation of possible visual and non-visual symptoms [13,14,15] (Table 1). Its prevalence is estimated to be 2.2% in the U.K. population [11]. The criteria for VSS include the following [1,13,14,15]: presence of VS for at least three continuous months; presence of two or more of the following visual symptoms: palinopsia, photosensitivity/photophobia, enhanced entoptic imagery, and impaired night vision (“nyctalopia”); and, in addition, individuals usually report one or more of the following visual and non-visual symptoms: photopsia, migraines, phonophobia, hyperacusis, cutaneous allodynia, tinnitus, balance problems, and tremor. Hence, those with VSS present with a wide range of sensory, motor, and perceptual symptoms. Based on these symptoms, and the earlier diagnostic literature criteria [1,2,3,4,5,6,11,12,13,14,15,16,17], a VSS rating-scale questionnaire was developed to assist in their clinical care [1]. Recently, a more detailed symptom questionnaire has been developed [18]. Thus, the use of these two different questionnaires provides a broad overview of the patient’s symptoms and provocative conditions.

A detailed review of the underlying neural substrate(s) for VS/VSS remains equivocal based upon the existing brain imaging studies [1]. Some of the neural sites likely to be involved include the right lingual gyrus, the visual cortex and its visual processing centers, the thalamocortical pathways, and the attentional/salience networks. Additional brain imaging studies are needed in this important area.

A common hypothesis for the presence of VS is “cortical hyperexcitability” [19,20], or the flip side, namely a reduced sensory threshold for perception [21]. Hence, one sees/perceives what others do not. However, another hypothesis has recently been proposed [22] that involves the extrastriate area of the middle temporal (MT) region. This area is involved in visual motion perception. While it normally has poor retinotopic representation, the small clusters of cells within it are, in fact, directionally-specific. If damaged by a head injury/concussion, spontaneous activity and hyperexcitability would occur. Such increased chronic activity would, in turn, produce current spread, which would lead to stimulation of large populations of these smaller regions, and produce multi-directional visual motion signals, thus representing the possible origin of the VS. Additionally, MT’s chromatic input could account for the chromatic aspect of the VS [23].

**Table 1:** (**A**) The table below categorizes and describes the primary visual symptoms reported in visual snow syndrome with percentages based on Tannen et al. (2022) [21]. (**B**) The table below categorizes and describes the reported secondary non-visual symptoms in visual snow syndrome with percentages based on Tannen et al. (2022) [21].

(A)			
Category	Percentage Reported *	Symptom	Description
**Visual**	100%	Visual Snow	Persistent grainy or pixelated visual disturbance across the entire field of vision, similar to TV static.
**Visual**	85%	Afterimages (Palinopsia)	Lingering visual traces of objects, sometimes with a trailing, comet-like effect.
**Visual**	74%	Light Sensitivity (Photosensitivity)	Discomfort or intolerance to bright lights or specific lighting conditions.
**Visual**	51%	Enhanced Entoptic Phenomena	Heightened perception of floaters or other ocular particles typically not visible.
**Visual**	33%	Night Vision Difficulty (Nyctalopia)	Impaired ability to see in low-light or night-time conditions.
**Visual**	30%	Flashes of Light (Photopsia)	Random, spontaneous perceptions of light flashes without external stimuli.
**(B)**			
**Category**	Percentage Reported *	**Symptom**	**Description**
**Non-Visual**	26%	Balance Issues	Sensations of physical unsteadiness or difficulty maintaining balance.
**Non-Visual**	15%	Sound Sensitivity (Hyperacusis)	Increased sensitivity to sounds, where normal noises feel overly intense.
**Non-Visual**	26%	Sound-Related Fear (Phonophobia)	Excessive fear or distress triggered by common environmental sounds.
**Non-Visual**	26%	Migraine Headaches	Severe, often one-sided headaches, potentially with nausea or temporary visual disturbances.
**Non-Visual**	4%	Tremors	Small, involuntary, rhythmic muscle contractions.
**Non-Visual**	48%	Ear Ringing (Tinnitus)	Persistent perception of ringing or buzzing in the ears without external cause.
**Non-Visual**	7%	Skin Sensitivity (Cutaneous Allodynia)	Pain or discomfort from light touch or normal skin contact.

* see ref [21].

## 2. Methods

In performing this review, a variety of search engines such as Pubmed, Google Scholar, and Semantic Scholar were used, as well as key words. In addition to peer-reviewed journal articles, related book chapters from excellent academic texts were consulted to provide foundational knowledge and context. All retrieved sources were critically evaluated by the authors for credibility, methodological rigor, and relevance to the focused review’s scope. However, there are limitations to a “narrative review.” First, it does not aim to be exhaustive, hence it is “focused.” Second, it does not follow a strict well-defined protocol. Third, it does not need to be reproducible, with much based on the author’s experience and expertise.

## 3. Treatment of VS/VSS

The treatment and overall management of VS/VSS has accelerated over the past decade [1,24]. This has resulted in six primary, non-mutually exclusive approaches based on the medical, bioengineering, and neuro-optometric literatures [1] (Table 2).

### 3.1. Discussion of the Condition with the Patient

The first therapeutic approach is the simplest, and yet very effective, in the initial stages of the case [21,25]. A casual discussion of the condition with the patient puts them at ease. They are told that it is not as rare as once believed, but more importantly, that in nearly all cases, it is benign. Such assurance is critical, as many also exhibit depression (~40%) and anxiety (~45%) [12,24,25]; they worry that the VS might worsen, which is unlikely, and that they may eventually become blind from it, which is not true. However, to err on the safe side, a referral to a neurologist is recommended for consultation and possible brain imaging. In addition, referral to either a psychiatrist or neuro-psychologist would be helpful for treatment of their general depression and anxiety, as well as for promotion of their overall well-being. In rare cases, the VS might be disease-related (e.g., Creutzfeldt–Jacob disease) [26,27]. If so, referral to an ophthalmologist or other medical specialist is also recommended.

### 3.2. Medications

The second approach has been the use of prescribed medications [1,28]. The three most prescribed include lamotrigine, an anti-convulsant; verapamil, an anti-hypertensive; and acetazolamide, a diuretic, with the first having benefits in a small number of these patients [24]. Since the physiological and biochemical mechanisms of VS are not yet well-defined [1,28], this has hindered the development of an effective, targeted treatment. For example, in the few studies conducted [1,18,24], the results have been mixed at best: typically, only 10–20% report partial remission of the VS. And, unfortunately, up to 40% have reported exacerbation of the VS [18]. Hence, at present, this is not the preferred treatment modality. More formal studies in the area need to be performed, and if successful, may provide the patient with a single and effective treatment.

### 3.3. Oculomotor-Based Vision Therapy

The third approach involves oculomotor remediation, that is, oculomotor-based vision therapy. The individual with VSS exhibits abnormalities of the sensory, perceptual, and motor systems [1,29]. This last category includes tremors and balance problems. However, more recently, it was discovered that other motor systems frequently (~60%) [21] manifest general problems of an oculomotor nature, including abnormalities of the vergence system used to track objects in depth to prevent diplopia; the versional system (e.g., saccades, pursuit) used to track objects laterally in different directions of gaze to prevent positional and velocity errors; and the accommodative system used to focus upon objects at different distances to prevent defocus blur [30]. These three systems function in a synkinetic manner to result in time-optimal, single and clear, binocular vision [30]. Any insult adversely affects this tightly-knit, temporal neurological arrangement [30]. The above is also consistent with laboratory reports of subtle oculomotor dysfunctions involving the saccadic system and its attentional component [31,32,33]. Hence, general motor and specific oculomotor problems appear to be prevalent in the VS population. Clinically, in the neuro-optometric diagnosis, this frequently includes convergence insufficiency, accommodative insufficiency, esophoria, saccadic dysmetria, and more [1,21]. Such dysfunctions frequently lead to oculomotor-based reading problems [1,21], typically resulting in reduced reading speed, rereading lines of print, and skipping lines, as well as transient blur and diplopia, all having an adverse impact on reading comprehension and visual comfort. Theses deficits have been documented using conventional optometric and ophthalmological clinical testing procedures (e.g., near point of convergence), specialized reading tests, and objectively-based assessment and quantification of the actual reading eye movements [1].

Fortunately, these oculomotor dysfunctions can be remediated with a series of well-documented, programmed eye movement training techniques [30]. This typically involves the saccadic, pursuit, fixation, vergence, and vestibular systems, with such therapy taking 12–24 sessions of a 45 min duration. The success rate has been excellent (~90%) [21]. The underlying neural mechanisms for the therapy incorporate the well-established scientific principles of perceptual and motor learning involving visual system plasticity [34]. However, there is a lack of formal clinical trials in this area with a large sample size and controls, which are needed to assess more critically the efficacy of the treatment modality. Moreover, better methods need to be developed to assess some of the symptoms such as palinopsia.

Serendipitously, it has been found that the aforementioned saccadic tracking component for the saccadic dysmetria also reduced the intensity and frequency of the problematic palinopsia [21]. It is believed that the specific, programmed saccadic training protocols function secondarily to “reset” the abnormal and reduced saccadic suppression threshold [21,30]. That is, it has been speculated that the normal saccadic suppression threshold used to prevent seeing afterimages and smeared retinal/cortical imagery (i.e., “trailing”) as one saccades is too low in those with VSS and palinopsia; thus, they “see/perceive” what others do not. This is consistent with the overall notion that in these individuals, their sensory systems are “hypersensitive” [1], which appears to reflect general, abnormally-low sensory thresholds, and hence results in these individuals perceiving images/sensations that are typically below the normal sensory–perceptual threshold. This general notion carries over to their other common problems, such as tinnitus, hyperacusis, cutaneous allodynia, photopsia, enhanced entoptic imagery, and photosensitivity. Thus, it appears to be an overall, unifying hypothesis involving global abnormally-reduced sensory thresholds in individuals with VSS.

### 3.4. Chromatic Tints

**Figure 3:**
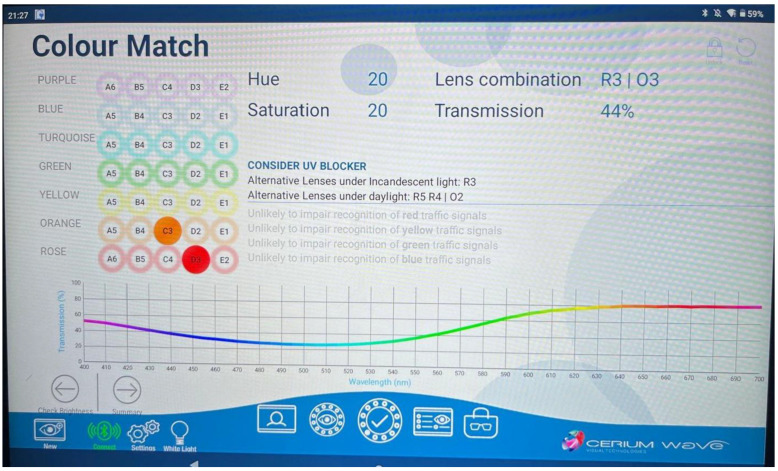
Intuitive Colorimeter (IC) tint specification (**Top**). Spectral range and magnitude of the effect with respect to transmission as a function of wavelength of a precision tint (**Bottom**).

One of the easiest and most highly successful treatments has included the use of special chromatic tints, typically in spectacle form incorporating the patient’s refractive correction [1,22,28,35,36]. This was first described in 2016 in a pilot study [37]. They tested 12 individuals with VS using the Intuitive Colorimeter (IC) [35] to ascertain the optimal precision chromatic tint. A total of 11 of the 12 reported some immediate degree of symptomatic relief (~92%) with the tint: the VS was reduced. Most (~83%) selected a tint/filter favoring/biased towards the blue–yellow spectral transmission region. Retesting yielded the same result. However, it was not further pursued by the investigators. Figure 3 shows the detailed IC computer output.

The next to pursue this promising therapeutic approach was the Ciuffreda group in 2019 in a pediatric (ages 10–15 years) case series (n = 3) [38]. First, they developed a VS/VSS symptom-rating scale questionnaire based on prior literature findings [1,2,3,4,5,6,11,12,13,14,15,17], as mentioned earlier. One patient reported VS immediately following removal of a brain cyst 2 years earlier. The medical examination was otherwise unremarkable. The VS was constant and usually monochromatic, but at times purplish-green in color. She also reported palinopsia when reading, general photosensitivity, transient episodes of photopsia, and some balance problems. Two prescriptions were prescribed: one reduced the VS by 75%, and the other reduced the palinopsia at near when reading by 65%. The other two cases had similar positive findings. In addition, one individual also received basic oculomotor training, which reduced her near-visual symptoms (e.g., blur at near), as well as the palinopsia, as described earlier. Thus, these three cases demonstrate the significant impact of a combination of special chromatic tints and oculomotor training on VS and palinopsia, as well as photosensitivity and photopsia; there was no effect on balance, however. Hence, the overall treatment plan markedly reduced all of the visual symptoms.

This was followed by a series of papers in a review by this same group [1]. In essence, the findings were similar in the majority of those with VS/VSS, with success in 80–90% of the patients, per remediation of most of the perceptual and motor symptoms. While there are no published studies on the long-term benefits of chromatic tints, our clinical experience shows a maintained effect over several years. However, additional studies by others as well as a randomized clinical trial (RCT) need to be conducted to more formally assess treatment efficacy. This remains an important current limitation.

This led to the development of a formal VS/VSS treatment protocol incorporating two main aspects: the VS/VSS and the binocular vision symptoms [35]. The first (Table 3) included the comprehensive vision assessment. In addition, there were three questionnaires: one for the VS/VSS, one for the oculomotor/binocular problems, and one for the photosensitivity, with all having rating scales for the quantification of symptom severity at baseline, and then again after some treatment. The second involved the actual VS/VSS assessment (Table 4). This included the use of the IC for ascertaining the optimal precision tint, use of readily available general ophthalmic chromatic tints, and also use of other special ophthalmic chromatic tints (i.e., FL-41 and BPI-Omega) (Colorlenses.com). Then, the effectiveness of this tint was assessed under an array of naturalistic conditions (e.g., computer scrolling and walking), with a follow-up, if a tint was prescribed. An example of the IC output for a precision tint is presented in Figure 3, which demonstrates its chromatically-biased spectral transmission (i.e., blue–yellow), as well as its overall reduced light transmission, which must be considered in the prescribing process, for example in those performing night-time driving in reduced illumination settings.

One recent theory has received considerable attention regarding the neural sites/pathways involved in the etiology of VS and the application of precision chromatic tints: disruption of the thalamocortical pathways [37,39]. Using the IC test instrument, they found that VS was reduced with precision chromatic filters in the orange–yellow and turquoise–blue spectra, but VS was exacerbated with filters in the blue–violet region. This latter finding suggested that S-cone activation, which initially inputs to the koniocellular layers and then inputs to the thalamocortical (TC) pathways, was the source. When the TC is disrupted, say by a concussion, oscillatory dysthymia/desynchronization occurs in the TC pathways involved in sensory perception and conscious awareness, hence the visibility of VS and the other related, abnormal perceptual sensory phenomenon (e.g., the palinopsia derived from the parietal cortex). This tentative theory warrants further laboratory testing using both brain imaging and visual psychophysical approaches.

### 3.5. Adaptation to Visual Noise

Recently, visual noise adaptation has been proposed for treatment [40]. It involves a classical, visual motion adaptation paradigm [41]. Basically, the individual with VS adapts to a dynamic, random visual noise motion stimulus for various periods of time (i.e., 5, 15, 45, and 135 s). Following the adaptation period, they then immediately report the magnitude of the perceived reduction in VS, as well as its duration. In a group of 25 individuals, the VS totally disappeared in fourteen, decreased in one, had no effect in six, worsened in two, and one reported other changes. Thus, 56% reported a temporary total disappearance of their VS, with it being the greatest for the longest duration of visual noise adaptation. However, on average, the positive post-effect lasted only 14 s. While intriguing, its short effect is disappointing. However, it may warrant further investigation in a larger test population, or perhaps as an adjunct treatment, in the future. While the aforementioned interpretation may be correct, there is an alternative one [42]. Assuming that VS represents pure random noise, and given that the adaptive stimulus is pure random noise, then if they are neurologically and mathematically averaged over a period of time, that is the external stimulus noise and the internal brain noise, the net result is to have effectively either zero, or reduced, noise. Hence, the transient reduction/disappearance of the human VS occurs for a short period of time. This too deserves further consideration using both laboratory testing and computer modelling of the phenomenon. For example, bioengineers need to be included in the development of this and other technology involving noise adaptation, as well a signal-to-noise analysis.

### 3.6. Environmental Alterations

The last treatment involves environmental changes/alterations. The perception of VS has been demonstrated to vary with illumination conditions [18]. It was the worst for outdoor night settings, and also problematic with fluorescent lights and interior lighting in general. It was least perceived outdoors in either sunny, cloudy, or rainy conditions. This information is helpful with respect to advice on VS expectations as one goes about their activities of daily living (ADL). The new questionnaire used by these investigators would help the clinician, and others, in providing general guidance for these patients. In another study [43], they suggested the use of LED lighting. Lastly, one could alter the paint color scheme of certain rooms to reduce VS perception. Further formal testing is warranted here. For example, a symptom-rating scale questionnaire should be developed to quantify any subjective improvement noted by the patient, and such information could be incorporated into color schemes for both the work environment and classroom.

## 4. Conclusions

VS/VSS represents a relatively new condition in clinical medicine. Fortunately, a key finding is that there are a number of current treatments (e.g., chromatic tints and oculomotor training), that reduce the perception of VS and related abnormal sensory/motor/perceptual phenomena (e.g., palinopsia). Additionally, treatment options will likely improve in the future (e.g., medications and visual noise adaptation). Lastly, considerable clinical and basic research in this exciting and new clinical medical frontier will likely continue, allowing clinicians to understand this unusual and complex neurological condition. For example, a randomized, clinical trial (RCT) for each of the current, and later future, treatment options needs to be conducted to assess, in an unbiased manner, the efficacy of a treatment in a large sample population with controls. In this regard, the clinical researcher and clinician need to work together to provide the best possible study, and outcome, for the patient. In addition, brain imaging studies should be performed with and without the therapeutic intervention to obtain objective data. Furthermore, this will allow one to identify related neural substrates in these still elusive medical conditions.

## Figures and Tables

**Table 2:** Treatment for visual snow syndrome.

Treatment	Details
Discussion of the condition with the patient	Educate patients regarding the typically benign nature Refer to psychology and neurology
Medications	- Lamotrigine - Verapamil - Acetazolamide
Oculomotor-based vision therapy	- Vergence - Version - Accommodation
Chromatic tints	- BPI Omega - FL-41 - Custom
Adaptation to visual noise	Short treatment periods
Environmental alterations	Lighting Painting scheme

**Table 3:** VSS comprehensive vision assessment. With permission, from [35]. Original Table 1.

**1.** **Detailed case history**	
	Basic case history
	b. Completion of VSSSS questionnaire
	c. Completion of BIVSS global symptom questionnaire
	d. Completion of VLSQ-8 photosensitivity questionnaire
**2.** **Comprehensive vision examination: refractive, binocular, ocular health**	
**3.** **Additional sensory testing**	
**4.** **Expanded binocular vision/oculomotor work-up**	

**Table 4:** Visual snow syndrome tint assessment and prescribing. With permission, from [35]. Original Table 2.

**1.** **Initial tint selection**	
	Intuitive Colorimeter
	b. Full spectrum of general ophthalmic tints
	c. FL-41 and BPI-Omega tints
**2.** **Performance-specific tint selection, including vision therapy for any binocular/oculomotor dysfunction, with chromatic tint if deemed beneficial from the above testing.**	
	Computer simulation of various environmental conditions
	b. Natural surroundings (especially provocative ones)
	c. Computer scrolling
	d. Different illuminations
	e. Long hallway with obstacles and signage
	f. Follow-up: 1, 3, and 6 months
